# Novel regulators of nucleic acid sensing drive neurotoxic interferon response in microglia

**DOI:** 10.1002/alz70855_099785

**Published:** 2025-12-23

**Authors:** Amanda McQuade

**Affiliations:** ^1^ UCSF, San Francisco, CA, USA

## Abstract

**Background:**

Large‐scale genetic studies and eQTL analyses have revealed microglia as critical players in Alzheimer's disease (AD). Consequently, there has been a deep focus on defining microglial activation states across models of AD. These studies have revealed several activation states to be enriched in AD including Interferon‐Responsive Microglia (IRM). IRM are hypothesized to represent a toxic activation state that promotes chronic neuroinflammation and loss of synapses. Thus, negative regulators of the IRM response are likely to be beneficial therapeutic agents to target microglial activation in AD.

**Methods:**

To uncover regulators of this maladaptive interferon‐response state, we performed a genome‐wide CRISPR interference screen for IFIT1 expression, a conserved marker of the interferon‐response state. Screening was performed in human iPSC‐derived microglia pre‐stimulated with IFNβ to enrich discovery of negative regulators of IRM which are proposed to slow tau accumulation and synaptic loss in AD.

**Results:**

These experiments uncovered both canonical regulators of interferon signaling as well as novel regulators of the IRM state including RNA processing and DNA methylation pathways. Importantly, these inhibitory effects were specific to regulation of the IRM state and did not broadly impair activation towards more beneficial disease response states. Further investigation into the mechanisms that underlie inhibition of interferon‐response have converged on nucleic acid sensing as a critically important regulator of sterile IRM.

**Conclusions:**

Mounting evidence shows that accumulation of cytoplasmic nucleic acids occurs as an early pathogenic marker of AD and that altering microglial responses to these disease‐associated molecular patterns can abrogate symptom onset in murine models. We have discovered novel regulators of nucleic acid sensing and the interferon‐responsive state using iPSC‐derived microglial models and CRISPR screening technologies. These findings provide a foundation to support development of targeted therapies that inhibit IRM in vivo and restore protective functions of microglia.

